# The Quality of School Wellness Policies and Energy-Balance Behaviors of Adolescent Mothers

**Published:** 2011-02-15

**Authors:** Debra Haire-Joshu, Byron W. Yount, Elizabeth L. Budd, Cynthia Schwarz, Rebecca Schermbeck, Scoie Green, Michael Elliott

**Affiliations:** Center for Obesity Prevention and Policy Research, George Warren Brown School of Social Work and School of Medicine, Washington University in St. Louis; Washington University in St. Louis, St. Louis, Missouri; Washington University in St. Louis, St. Louis, Missouri; Washington University in St. Louis, St. Louis, Missouri; Washington University in St. Louis, St. Louis, Missouri; Washington University in St. Louis, St. Louis, Missouri; Washington University in St. Louis, St. Louis, Missouri

## Abstract

**Introduction:**

In this study, we 1) compared the quality of school wellness policies among schools participating in Moms for a Healthy Balance (BALANCE), a school- and home-based weight loss study conducted with postpartum adolescents in 27 states; and 2) assessed the relationship between policy quality with energy-balance behaviors and body mass index *z* scores of postpartum adolescents.

**Methods:**

As a part of BALANCE, we collected data on high-calorie food and beverage consumption, minutes spent walking, and height and weight for 647 participants. The School Wellness Policy Coding Tool was used to assess the strength and comprehensiveness of school district wellness policies from 251 schools attended by participating adolescent mothers.

**Results:**

Schools averaged low scores for wellness policy comprehensiveness and strength. When compared with participants in schools with the lowest policy comprehensiveness scores, adolescent mothers in schools with the highest scores reported consuming significantly fewer daily calories from sweetened beverages while reporting higher consumption of water (*P* = .04 and *P* = .01, respectively). School wellness policy strength was associated with lower BMI *z* scores among adolescent mothers (*P* = .01).

**Conclusion:**

School wellness policies associated with BALANCE may be limited in their ability to promote a healthy school environment. Future studies are needed to evaluate the effect of the strength and comprehensiveness of policy language on energy balance in high-risk postpartum adolescents. Evidence from this work can provide additional guidance to federal or state government in mandating not only policy content, but also systematic evaluation.

## Introduction

Approximately 18% of adolescents aged 12-19 years or 9 million youth in the United States are overweight (1). The risk of overweight is significantly heightened for the approximately 500,000 adolescents who become pregnant each year (2). Postpartum weight retention exacerbates the risk of development of overweight, impaired glucose tolerance, type 2 diabetes, and other diseases (3-7). Strategies addressing high-risk patterns among adolescent mothers may have important public health implications, as postpartum weight retention may compound with future pregnancies and timely interventions may mitigate the intergenerational transfer of high-risk behaviors (4,8).

Environmental and policy interventions for food and activity environments may be effective strategies for preventing childhood obesity (9). Policy interventions create population access to environments that promote healthy options (10,11). Some policy initiatives have targeted schools (12). Children may spend up to 10 hours per day at school, which accounts for much of their physical activity and as many as 2 meals and 2 snacks per day. The Child Nutrition and WIC (Women, Infants, and Children) Reauthorization Act of 2004 (Public Law 108-265), which went into effect in 2006-2007, required all local education agencies participating in the National School Lunch Program to create a school wellness policy that included goals for achieving energy balance through healthy dietary intake and physical activity behaviors (13-15).

To date, preliminary data have shown mixed results regarding the quality of school wellness policies (12). Variations in measures used in evaluating policies make interpretation of findings challenging and limit the opportunity for comparative analyses of school wellness policies across communities and states (16,17). Schwartz and colleagues (18) developed a measure to evaluate the quality of school wellness policies across common criteria for comprehensiveness (ie, breadth of areas covered) and strength (ie, degree to which policies included specific and firm language).

In this study, we 1) compared the quality of school wellness policies of schools participating in Moms for a Healthy Balance (BALANCE) (19), a school- and home-based weight loss study conducted with postpartum adolescents across 27 states; and 2) assessed the relationship between policy quality with energy-balance behaviors and body mass index (BMI) *z* scores of postpartum adolescents.

## Methods

### Design and sample

BALANCE was a group-randomized, nested-cohort study developed and designed in partnership with Parents As Teachers (PAT), a national parenting and child development program (20). We recruited postpartum adolescents who retained their pregnancy weight to participate in the BALANCE weight-reduction protocol. We used data from BALANCE baseline assessments that participants completed between January 2007 and April 2008. As part of our BALANCE study during 2008-2009, we collected school wellness policies from the websites of schools or school districts attended by our participants. If the policy was unavailable on the website, we contacted the school and requested a copy. We also verified that collected policies were in effect in 2006-2007.

We recruited 1,330 ethnically diverse participants into BALANCE who were enrolled in PAT Teen Parent Programs from 27 states (Appendix A). In addition to enrollment in the PAT Teen Parent Program (for ages 13-19 y), eligibility criteria included 1) a willingness to participate throughout the study period, 2) being less than 1 year postpartum, and 3) not being pregnant or planning to become pregnant during the study period. For our analysis, we further excluded participants who had either graduated or withdrawn from school (n = 275), were currently breastfeeding (n = 109), or were missing residential zip code and school information (n = 299). In total, 647 postpartum adolescents located in 251 schools from 203 school districts in 27 states, contributed to our findings. The institutional review board of Washington University in St. Louis approved this study.

Our sample had a mean age of 17.2 (standard deviation 1.1 y). Forty-eight percent were white, 30% were black, and 22% were other; most were receiving some form of aid from either WIC (91%) or the federally sponsored free or reduced-lunch program (40%), and they were approximately 6 months postpartum (182 days). Approximately half of participants were at a normal weight and half were overweight or obese.

### Measures

Participants' height and weight were collected by trained PAT staff to determine BMI *z *score classification according to criteria specified for adolescents by National Health and Nutrition Examination Survey procedures (21).

Adolescents then completed the online Snack and Beverage Food Frequency Questionnaire (SBFFQ), which was used to measure specific high-calorie snack and beverage consumption patterns of participants. Following a similar format to that of the Diet History Questionnaire (22), the SBFFQ examined each participant's intake of 31 items during the previous 7 days by asking on how many days, how many times per day, and how much of the item the participant consumed. Food items were assessed by subgroups: sweetened beverages (eg, soda and fruit juice), salty snacks (eg, potato chips), sweet snacks (eg, hard candy), meal-type snacks (eg, french fries), fruits and vegetables, and water consumption. Intake was converted into the total calories consumed for each individual food item and summed to obtain the daily calorie total. The test–retest reliability for the separate measures ranged from moderate to substantial with the following intraclass correlation coefficients: water (.71), sweetened beverages (.68), salty snacks (.43), meal-type snacks (.64), and fruits and vegetables (.46) (23). The test–retest reliability for the composite measure of total calories was acceptable (.63).

Physical activity was measured with 3 items asking participants how many minutes they spent walking at a slow, brisk, or very brisk pace on the 2 weekdays preceding completion of the measure, and on 1 weekend day (24). Participants reported their age, race/ethnicity, education level, breastfeeding status, and postpartum status. They also reported their participation in aid programs (WIC and the National School Lunch Program), which we used as indicators of socioeconomic status.

We used the 96-item School Wellness Policy Coding Tool developed by Schwartz and colleagues (18) to assess the strength and comprehensiveness of the school wellness policies in each school district (Appendix B). Each of the 96 content items was coded with a score of 0, if the item was not mentioned; 1, if the item was a "weak" statement making it hard to enforce because of vague, unclear, or confusing language; or 2, meaning the item "meets or exceeds expectations" since it was mentioned in a specific and directive manner suggesting commitment to enforcement (Table 1).

### Data analysis

Data analyses were conducted in 2 stages. First, we sought to determine the comprehensiveness and strength scores of school wellness policy language for school districts attended by our participants. Second, we sought to relate the overall comprehensiveness and strength of school wellness policy language to the measured energy-balance behaviors and BMI *z* scores of BALANCE participants. All analyses were conducted by using SPSS version 17.0 (SPSS, Inc, Chicago, Illinois).

We evaluated the language quality for each policy item of the coding tool by the percentage of school districts with a rating of "meets or exceeds expectations." For assessing the language quality of the 7 policy sections and the overall district policy score, we computed the sample mean and standard deviation for both comprehensiveness and strength with methods suggested by Schwartz and colleagues (18).

The school wellness policy language scores for both comprehensiveness and strength were split into low, middle, and upper tertiles. We compared demographic characteristics of BALANCE participants among school wellness policy language tertiles with χ^2^, Kruskal-Wallis, or 1-way analysis of variance tests, as indicated by measurement level. Univariate, general linear models were constructed to assess the relationship between school wellness policy comprehensiveness and strength tertiles and measured energy-balance behaviors. We explored the possibility that relationships between policy language quality and energy-balance behaviors may vary by either race/ethnicity or BMI, by testing the race/ethnicity × policy score tertile and BMI × policy score tertile cross-product terms. Final models were adjusted for race/ethnicity, as both the scoring of policy quality and energy consumption of snacks appeared to vary by race/ethnicity in our sample. The statistical assumptions underlying each test were checked for violations (eg, homogeneity of variances and outlying and influential cases). Given that our sample had little variation regarding school wellness policy comprehensiveness or strength scores, we selected the 40 highest and 40 lowest scoring districts for further analysis.

## Results

### District school wellness policies

Appendix B displays the 96 items measured by the policy coding tool and the percentage of districts with policies that received a rating of 2 (meets or exceeds expectations). In general, federally mandated statements accounted for a high percentage of items that met or exceeded expectations in each section. Five school districts had policies that did not address any of the 7 sections of the policy coding tool. The section that received the highest number of zero ratings was nutrition standards for competitive and other foods and beverages (n = 101 school districts); the least number of zero ratings was for standards for US Department of Agriculture child nutrition programs and school meals (N = 16 school districts).

### Relationship of policy quality to dietary intake, physical activity, and BMI *z *score

When assessed for group differences across tertiles of school wellness policy comprehensiveness and strength scores, race/ethnicity and BMI *z* score were unbalanced (Table 2). Specifically, white mothers were more commonly found in districts with the highest policy rating, while black mothers were more commonly found in districts with the lowest policy rating. Additionally, the lower tertiles of both comprehensiveness and strength scores included adolescents with higher BMI *z *scores, though the group comparison was not significant for policy strength. We found no evidence of effect modification for either race/ethnicity or BMI when considering the relationship between school wellness policy quality and energy-balance behavior outcomes. In our initial adjusted models assessing snack and physical activity behaviors of participants, we found no significant relationships between policy comprehensiveness or strength tertile and energy-balance behaviors.

In the 40 school districts that had the highest scores for policy comprehensiveness, adolescent mothers reported consuming fewer daily calories from sweetened beverages and more water (Table 3). There was an inverse relationship between policy comprehensiveness and strength and salty, sweet, and meal-type snacks and total snack calories. Policy strength was significantly associated with a lower BMI *z* score and was also inversely related to sweetened beverage consumption.

## Discussion

Four findings from this study can expand research related to policy initiatives associated with promoting energy-balance behaviors among adolescent mothers. First, our study suggests that items that are mandated in school wellness policies are most likely to meet or exceed expectations for quality language when compared with nonmandated items. This study also supports previous studies that have found that strong mandatory language, as opposed to recommended language, has the greatest effect on food access (12,25). Clarification of school wellness policy language by the federal government to address both strength and scope of content may further enhance the effect of school wellness policies for adolescent mothers. State governments have the best knowledge of needs, possible incentive programs, and the financial situation of their state when crafting the model policies for school districts.

Second, our study suggests there are differences in the quality of policies that have an educational focus compared with those focused on behavioral outcomes. Previous studies have reported variations in the extent to which nutrition or physical activity topics are included in school wellness policies (26). We were able to expand on this work and systematically measure and compare both the comprehensiveness and strength of nutrition and physical activity focused topics in policies among multiple states and school districts (18). Of particular note was that 2 of the sections (establishing nutrition standards for competitive and other foods and beverages, physical education) requiring language for policy actions directly related to regulating food access and time to be physically active scored the lowest for comprehensiveness and strength. In contrast, sections scoring the highest included evaluation and nutrition education, which each focused on establishing goals or documenting a plan for implementation as opposed to mandating immediate changes in the environment (25,27,28). Further study is warranted to describe reasons for these differences, barriers to the development and implementation of strong and comprehensive policies, and the extent to which they may affect behavior (9,29-31).

Third, our study found that schools associated with PAT programs for adolescent parents have generally weak wellness policies in place. Additionally, there appeared to be a relationship between the presence of weak policies and energy-balance behaviors of adolescent mothers. For example, the most comprehensive policies were associated with adolescent mothers consuming 136 fewer calories from sweetened drinks per day and by 17 ounces more water per day. Indeed, substantial literature suggests a relationship of sweetened beverages to obesity (32,33). Others have also found sweetened beverage intake was altered by school environmental changes (11,14). Our study contributes to this literature by further suggesting the value of policy quality in addressing beverage intake in schools as a possible mechanism for preventing obesity.

Finally, from a translational perspective, our findings suggest the importance of defining the model content of quality school wellness policies, and effectively communicating to parents as to whether this content is present in school policies (9,34,35). Currently, adolescents or their parents have no way of adequately judging the quality of the wellness policy that directly influences the school environment. The overwhelming presence of weak school wellness policies might mislead parents or adolescents into thinking their educational environment practices and reinforces positive eating and activity behaviors. Our results, consistent with those of other studies, suggest that in fact this may be the case (12,26). Wellness report cards or other strategies for communicating the strength and comprehensiveness of school policies to parents in easily understandable ways are needed (36-38).

### Limitations

Our study had several limitations. First, this is a cross-sectional study that does not allow for assessment of temporal relationships. We did not assess policy effect or implementation which may vary by school district. We had limited information on the school districts in our sample, so were unable to address heterogeneity and generalizability issues. Many of the policies under observation were not required until the start of the 2006-2007 academic year, which may not provide enough time to see the full effect of the policies on measured behaviors. We also present information on a group that may not be generalizable to broader school-district populations. Finally, interpretation of our findings should be considered within the limitations of self-report measures.

### Conclusion

School wellness policies associated with PAT programs for adolescent mothers in multiple states may be limited in their ability to promote a healthy school environment. Improvements in the quality of school wellness policies may help to enhance the school environment and, in turn, energy-balance behaviors of adolescents. Future studies, reflecting naturalistic or prospective designs, are needed to evaluate the effect of the strength and comprehensiveness of policy language on energy balance in high-risk postpartum teenagers. Evidence from this work can provide additional guidance to federal or state government in mandating not only policy content, but systematic evaluation. To be active advocates for their adolescent, parents need to be accurately informed about the quality of the wellness policies in their adolescent's school. Quality assurances are needed so that school wellness policies are not missed opportunities for encouraging energy-balance behaviors and preventing obesity among adolescent mothers.

## Figures and Tables

**Table 1:** School Wellness Policy Comprehensiveness and Strength Scores, 2007-2009

**School Wellness Policy Coding Tool^a^**	**Mean Score (SD)**	**Maximum^b^**
**Section 1: Nutrition education**		
Comprehensiveness score^c^	49.0 (28.8)	100.0
Strength score^d^	31.3 (22.2)	77.8
**Section 2: Standards for USDA child nutrition programs and school meals**		
Comprehensiveness score^c^	39.0 (24.3)	92.3
Strength score^d^	24.3 (17.5)	69.2
**Section 3: Nutrition standards for competitive and other foods and beverages**		
Comprehensiveness score^c^	37.7 (19.1)	72.4
Strength score^d^	9.3 (14.4)	58.6
**Section 4: Physical education**		
Comprehensiveness score^c^	32.5 (22.7)	88.2
Strength score^d^	17.8 (14.0)	52.9
**Section 5: Physical activity**		
Comprehensiveness score^c^	37.9 (27.3)	100.0
Strength score^d^	21.3 (18.3)	70.0
**Section 6: Communication and promotion**		
Comprehensiveness score^c^	39.5 (24.7)	100.0
Strength score^d^	21.0 (17.9)	66.7
**Section 7: Evaluation**		
Comprehensiveness score^c^	59.0 (25.5)	100.0
Strength score^d^	31.6 (25.9)	83.3
**Overall**		
Comprehensiveness score^e^	39.6 (19.1)	72.9
Strength score^f^	19.0 (12.6)	51.0

Abbreviations: SD, standard deviation; USDA, US Department of Agriculture.

a School Wellness Policy Coding Tool consists of 96 items split among 7 sections. Each item is rated 0 if the policy item was not mentioned, 1 if the policy item was written in vague or confusing language, or 2 if the policy item used specific and directive language.

b The minimum score for each tool was 0, and the maximum score was the highest score received.

c Comprehensiveness scores represent items rated either 1 or 2 within a section divided by the number of items in that section, indicating the policy addressed the section.

d Strength scores represent items rated a 2 within a section divided by the number of items in that section, indicating the policy addressed the section with specific and directive language.

e Overall comprehensiveness score is the average of the 7 comprehensiveness section scores.

f Overall strength score is the average of the 7 strength section scores.

**Table 2:** Characteristics of 647 Postpartum Adolescents, by School Wellness Policy Score Tertiles, 2007-2009

Measure	School Wellness Policy Score Tertiles							

	Comprehensiveness Score, Mean (SD)^a^			*P* Value	Strength Score,Mean (SD)^b^			*P* Value
	
	Lower	Middle	Upper		Lower	Middle	Upper	
**School level (n = 251)**								
**National School Lunch Program participants, %**	43.5 (22.2)	44.2 (21.2)	39.4 (17.4)	.11^c^	45.8 (21.8)	42.5 (22.0)	39.6 (17.5)	.02^c^
**Individual level (n = 647)**								
**Age, y (SD)**	17.2 (1.1)	17.2 (1.1)	17.2 (1.2)	.90^d^	17.2 (1.0)	17.3 (1.1)	17.1 (1.2)	.19^d^
**Race/ethnicity, %**								
White	44.5	44.3	57.1	<.001^e^	43.8	51.0	50.2	.004^e^
Black	41.6	27.1	22.2		40.4	26.3	25.1	
Other	13.9	28.5	20.7		15.8	22.7	21.2	
**BMI *z* score (SD)**	0.51 (1.1)	0.36 (1.0)	0.20 (1.2)	.03^d^	0.51 (1.1)	0.27 (1.1)	0.30 (1.2)	.08^d^
**Postpartum duration, d (SD)**	189.8 (97.4)	187.7 (97.7)	167.8 (98.6)	.08^d^	187.5 (96.3)	186.1 (95.4)	173.8 (101.7)	.35^d^
**WIC participants, %**	94.4	88.8	89.1	.08^e^	93.8	91.1	87.6	.08^e^

Abbreviations: SD, standard deviation; BMI, body mass index; WIC, Child Nutrition and Women, Infants, and Children Reauthorization Act of 2004.

a Comprehensiveness scores indicate common criteria for the breadth of areas covered by school wellness policies. Strength scores indicate the degree to which policies included specific and firm language. Minimum and maximum scores: lower, minimum = 0, maximum = 25; middle, minimum = 26.04, maximum = 47.92; upper, minimum = 48.96, maximum = 72.92.

b Strength scores indicate the degree to which policies included specific and firm language. Minimum and maximum scores: lower, minimum = 0, maximum = 10.42; middle, minimum = 26.04, maximum = 47.92; upper, minimum = 48.96, maximum = 72.92.

c Calculated by using the Kruskal-Wallis test.

d Calculated by using 1-way analysis of variance.

e Calculated by using the *χ^2^* test.

**Table 3:** Energy-Balance Behaviors of 647 Postpartum Adolescents, Bottom 40 and Top 40 School Wellness Policy Scores, 2007-2009

Characteristic	District Rating					

	**Comprehensiveness Score,** Mean (95% CI)^a^			**Strength Score,** Mean (95% CI)^b^		

	Bottom 40	Top 40	*P* V**alue**	Bottom 40	Top 40	*P* Value
BMI *z* score^c^	0.51 (0.23-0.79)	0.40 (0.14-0.66)	.56	0.55 (0.32-0.76)	0.14 (0.09-0.37)	.01
Water consumption, oz^c^	24 (17-32)	41 (34-47)	.01	28 (22-35)	36 (30-43)	.07
Sweetened beverage, kcal^c^	508 (405-610)	372 (285-460)	.04	421 (342-498)	372 (297-446)	.36
Salty snack, kcal^c^	369 (241-496)	376 (267-485)	.93	317 (222-411)	306 (216-396)	.86
Sweet snack, kcal^c^	268 (197-338)	277 (217-338)	.83	283 (228-337)	255 (203-307)	.46
Meal-type snack, kcal^c^	237 (158-315)	276 (209-343)	44	277 (217-338)	254 (196-311)	.56
Fruit and vegetable snack, kcal^c^	51 (30-74)	46 (27-65)	.69	39 (23-55)	47 (32-63)	.45
Total snack, kcal^c^	1,786 (1,523-2,050)	1,707 (1,481-1,933)	.64	1,699 (1,503-1,896)	1,568 (1,381-1,755)	.32
Walking, min	14 (10-18)	17 (13-21)	.25	16 (13-20)	17 (13-20)	.90

Abbreviations: CI, confidence interval; BMI, body mass index.

a Minimum and maximum scores: bottom 40 districts, minimum = 0, maximum = 19.79; top 40 districts, minimum = 61.46, maximum = 79.92.

b Minimum and maximum scores: bottom 40 districts, minimum = 0, maximum = 6.25; top 40 districts, minimum = 33.33, maximum = 51.04.

c General linear models adjusted for race/ethnicity.

## Appendices

### Appendix A

**Table:** States Where Postpartum Adolescents Participating in Moms for a Healthy Balance Reside

Region	**State**
South	Alabama, Arkansas, Florida, Georgia, Kentucky, Louisiana, Maryland, Mississippi, North Carolina, Oklahoma, South Carolina, Tennessee, Texas
Midwest	Illinois, Indiana, Iowa, Kansas, Michigan, Missouri, Ohio, South Dakota, Wisconsin
Northeast	New York, Pennsylvania, Rhode Island
West	Arizona, California

### Appendix B

**Table:** Percentage of School Districts (N = 203) That Meets or Exceeds Expectations for Each Item of the School Wellness Policy Coding Tool

Item	%
**Nutrition education**	
1. Includes goals for nutrition education that are designed to promote student wellness in a manner that the local education agency determines is appropriate (*federal requirement*)	76.8
2. Nutrition curriculum provided for each grade level	46.3
3. Coordinates nutrition education with the larger school community	19.2
4. Nutrition education extends beyond the school environment	14.3
5. District provides nutrition education training for all teachers	7.9
6. Nutrition education is integrated into other subjects beyond health education	23.2
7. Nutrition education teaches skills that are behavior-focused and/or interactive and/or participatory	35.0
8. Specifies number of nutrition education courses or contact hours	1.5
9. Nutrition education quality is addressed	57.6
**Standards for US Department of Agriculture (USDA) child nutrition programs and school meals**	
10. Assures guidelines for reimbursable school meals shall not be less restrictive than USDA school meal regulations (*federal requirement*)	89.7
11. Addresses access to and/or promotion of the USDA School Breakfast Program	6.9
12. Addresses access to and/or promotion of the Summer Food Service Program	3.4
13. Addresses nutrition standards for school meals beyond USDA (National School Lunch Program/School Breakfast) minimum standards	23.6
14. Specifies use of low-fat versions of foods and/or low-fat methods for preparing foods	8.9
15. Specifies strategies to increase participation in school meal programs	10.8
16. Optimizes scheduling of meals to improve student nutrition	11.8
17. Ensures adequate time to eat	18.7
18. Addresses access to hand washing before meals	25.6
19. Requires nutrition qualifications of school food service staff	23.2
20. Ensures training or professional development for food service staff	29.1
21. Addresses school meal environment	52.7
22. Nutrition information for school meals (eg, calories, saturated fat, sugar) is available	10.8
**Nutrition standards for competitive and other foods and beverages**	
23. Includes nutrition guidelines for ALL foods available on school campus during the school day with the objective of promoting student health and reducing childhood obesity (*federal requirement*)	35.5
24. Regulates vending machines	21.7
25. Regulates school stores	21.2
26. Regulates food service à la carte	22.2
27. Regulates food served at class parties and other school celebrations	2.0
28. Regulates food from home for the whole class	5.4
29. Regulates food sold before school	2.0
30. Regulates food sold after school that is not part of a district-run after-school program	1.0
31. Regulates food sold at evening and community events on school grounds	9.4
32. Regulates food sold for fundraising	17.2
33. Addresses limiting sugar content of foods	7.4
34. Addresses limiting fat content of foods	14.3
35. Addresses limiting sodium content of foods	3.9
36. Addresses limiting calorie content per serving size of foods	3.4
37. Addresses limiting serving size of foods	11.8
38. Addresses increasing whole foods (eg, whole grains, unprocessed foods, fresh produce)	1.5
39. Addresses limiting the use of ingredients with questionable health effects in food or beverages (eg, artificial sweeteners, processed or artificial foods, trans fats, high fructose corn syrup)	1.0
40. Addresses food not being used as a reward and/or withheld as a punishment	24.6
41. Nutrition information (eg, calories, saturated fat, sugar) available for foods other than school meals	2.0
42. Addresses limiting sugar content of beverages	13.3
43. Addresses limiting fat content of drinks (other than milk)	0.5
44. Addresses limiting calorie content per serving size of beverages	2.5
45. Addresses limiting regular (sugar-sweetened) soda	16.7
46. Addresses limiting beverages other than soda containing added caloric sweeteners such as sweetened teas, juice drinks, energy drinks, and sports drinks	1.5
47. Addresses limiting sugar/calorie content of flavored milk	15.3
48. Addresses limiting fat content of milk	1.0
49. Addresses serving size limits for beverages	3.9
50. Addresses limiting caffeine content of beverages (with exception of trace amounts of naturally occurring caffeine substances)	2.0
51. Addresses access to free drinking water	15.3
**Physical education (PE)**	
52. Addresses PE curriculum for each grade level	51.7
53. Addresses time per week of PE for elementary school students	15.8
54. Addresses time per week of PE for middle school students	12.3
55. Addresses time per week of PE for high school students	3.9
56. PE promotes a physically active lifestyle	45.8
57. Specifies competency assessment (ie, knowledge, skills, and practice)	6.9
58. Addresses PE quality	63.1
59. PE promotes inclusive play	4.4
60. Addresses PE classes or credits	7.4
61. Addresses frequency of required PE (daily)	1.5
62. Addresses teacher–student ratio for PE	2.0
63. Addresses safe and adequate equipment and facilities for PE	16.3
64. Addresses amount of time devoted to moderate to vigorous activity in PE	5.9
65. Addresses qualifications for PE instructors	32.0
66. District provides PE training provided for teachers	24.1
67. Addresses PE waiver requirements (eg, substituting PE requirement with other activities)	8.9
68. Requires students to participate in an annual health assessment (eg, fitness or body mass index)	1.0
**Physical activity (PA)**	
69. Includes goals for PA that are designed to promote student wellness in a manner that the local education agency determines is appropriate *(federal requirement)*	60.1
70. PA provided for every grade level	33.0
71. Includes PA opportunities for school staff	8.4
72. Regular PA opportunities are provided throughout the school day (not including recess)	8.4
73. Addresses PA through intramurals or interscholastic activities	26.6
74. Addresses community use of school facilities for PA outside of the school day	13.3
75. Addresses safe active routes to school	9.4
76. Addresses not using PA (extra or restricted) as punishment	25.6
77. Addresses recess frequency or amount in elementary school	23.6
78. Addresses recess quality to promote PA	4.9
**Communication and promotion**	
79. Involves parents, students, and representatives of the school food authority, the school board, school administrators, and the public in the development of the school wellness policy *(federal requirement)*	43.8
80. Includes staff wellness programs specifically addressing the health of staff	17.7
81. Addresses consistency of nutrition messages	25.1
82. Encourages staff to role model healthy behaviors	30.0
83. Specifies who in the district is responsible for wellness/health communication beyond required policy implementation reporting	7.9
84. Specifies district use of the Centers for Disease Control and Prevention's Coordinated School Health model or other coordinated/comprehensive method	1.5
85. Addresses methods to solicit or encourage input from stakeholder groups (eg, 2-way sharing)	17.7
86. Specifies how district will engage parents or community to meet district wellness goals	21.7
87. Specifies what content/information district communicates to parents	25.6
88. Specifies marketing to promote healthful choices	23.2
89. Specifies restricting marketing of unhealthful choices	11.8
90. Establishes a health advisory committee or school health council that is ongoing beyond policy development	26.1
**Evaluation**	
91. Establish a plan for measuring implementation of the local wellness policy, including designation of 1 or more persons within the local educational agency or at each school, as appropriate, charged with operational responsibility for ensuring that the school meets the local wellness policy *(federal requirement)*	65.0
92. Addresses a plan for policy implementation, including a person or group responsible (initial or ongoing)	36.5
93. Addresses a plan for policy evaluation, including a person/group responsible for tracking outcomes	16.7
94. Addresses the audience and frequency of a report on compliance and/or evaluation	40.4
95. Identifies funding support for wellness activities or policy evaluation	1.0
96. Identifies a plan for revising the policy	30.0
